# PROTOCOL: Intergenerational interventions and their effect on social and mental wellbeing of both children and older people—A mapping review and evidence and gap map

**DOI:** 10.1002/cl2.1235

**Published:** 2022-05-14

**Authors:** Joanna Thompson‐Coon, Fiona Campbell, Anthea Sutton, Rebecca Whear, Morwenna Rogers, Jane Barlow, Ellie Robinson Carter, Richard Sharpe, Stuart Cohen, Louise Wolstenholme

**Affiliations:** ^1^ NIHR ARC South West Peninsula (PenARC) University of Exeter Medical School Exeter UK; ^2^ School of Health and Related Research The University of Sheffield Sheffield UK; ^3^ University of Sheffield Sheffield UK; ^4^ NIHR CLAHRC South West Peninsula (PenCLAHRC) University of Exeter Medical School Exeter UK; ^5^ NIHR PenCLAHRC, Institute of Health Research University of Exeter Medical School Exeter UK; ^6^ University of Oxford Coventry UK; ^7^ Sensory Trust Fig Leaf Building Eden Project Bodelva Cornwall UK; ^8^ Public Health, Cornwall Council & University of Exeter Medical School St. Austell UK; ^9^ NHS Kernow Clinical Commissioning Group St. Austell UK; ^10^ 0‐19 Services Sheffield Childrens Sheffield UK

## Abstract

This is the protocol for a Campbell systematic review. The objectives are as follows: identify, appraise and bring together the evidence on the use of intergenerational practice.

## 1 BACKGROUND

### 1.1 Introduction

#### 1.1.1 The problem, condition or issue

Opportunities for social connection between generations in the United Kingdom have diminished over the last few decades because of changes in the way that we live and work (Kingman, 2016; United for all Ages, 2017). Housing and economic trends have seen younger people move to live in city centres whilst the older generation live in towns and rural areas. A report published by the Intergenerational Foundation in 2016 Kingman, 2016 suggests that in the 25 biggest cities within the United Kingdom only 5% of people aged over 65 live in the same neighbourhood as someone under the age of 18. Furthermore, even when people from different age groups do live in the same area, the decline in spaces such as libraries, youth clubs and community centres mean that there are fewer opportunities to meet and mix socially with other generations outside our own families. Increased working hours, improved technology, changes in family patterns, relationship breakdowns within families and migration are also believed to be contributory factors to generation segregation (Generations Working Together, 2019). There are many potential economic, social and political impacts of generations living separate and parallel lives, for example, higher health and social care costs, an undermining of trust between generations (Brown & Henkin, 2014; Jones, 2011; Laurence, 2016; Vitman et al., 2013), reduced social capital (Laurence, 2016), a reliance on the media to form understanding of others' viewpoints (Edström, 2018; Vasil & Wass, 1993) and higher levels of anxiety and loneliness. Loneliness is a huge issue in the United Kingdom and one that is shared by both the young and the old. In the Office for National Statistics Community Life Survey, 2016 to 2017 (ONS, 2021), 5% of adults in the United Kingdom felt lonely often or always and compared with all other age groups except the 25–34 years group. Those aged 16–24 were also significantly more likely to report feeling lonely often or always.

#### 1.1.2 The intervention

Intergenerational programmes and activities can take many formats and are delivered in many settings. Many are provided by third sector organisations. Although evidence suggests that intergenerational activity can have a positive impact on participants (e.g., to reduce loneliness and exclusion (for both older people and children and young people), improve mental health, increase mutual understanding and tackle important issues such as ageism, housing and care), commissioning decisions are complex due to the apparent wealth of options available.

#### 1.1.3 Why it is important to develop the EGM

Intergenerational programmes and activities may be promising interventions that can address some of the needs of both children and young people and older people. These interventions can take many formats and are delivered in diverse settings, often by third sector organisations. Although, evidence suggests that intergenerational activity can have a positive impact on participants commissioning decisions are complex due to the lack of evidence regarding which programmes to commission.

This evidence and gap map (EGM) will identify the nature, volume and types of interventions that have been undertaken and evaluated. It will identify areas for future research and evidence synthesis.

## 2 OBJECTIVES

We aim to use existing evidence to improve understanding of the role of intergenerational activities in health and social care from the perspectives of older people.

Our objectives are to:

– Identify, appraise and bring together the evidence on the use of intergenerational practice

To answer the following specific research questions:

What is the volume, nature and diversity of research on, and evaluation of, intergenerational practice and learning?
What approaches have been used to deliver intergenerational activities and programmes which may be relevant to providing such services during and in the subsequent recovery from the COVID‐19 pandemic?
What promising intergenerational activities and programmes have been developed and are being used but have not yet been subject to formal evaluation?

## 3 METHODS

### 3.1 EGM: Definition and purpose

EGMs are maps of a specific sector or subsector which typically includes both systematic reviews and primary studies. Produced using the same systematic approach as systematic reviews, both EGMs a usually show what evidence is there, not what the evidence says (White et al., 2018).

### 3.2 Framework development and scope

The framework will be developed with our stakeholders and will take into account exisiting frameworks as described below in the ‘conceptual frameworks’ section and including the Depth of Intergenerational Engagement Scale (Kaplan, 2004).

The scope of this EGM is to capture the broad range of evidence from systematic reviews and primary research that has investigated intergenerational practice.

The EGM will enable policymakers and practitioners in the field to take account of the least biased and most scientifically rigorous evidence in the commissioning and use of intergenerational practice in health and social care. It will also highlight opportunities for intergenerational activities and programmes during and in the subsequent recovery from the COVID‐19 pandemic and direct the commissioning of appropriate research where there are evidence gaps.

The scope of the EGM is defined by a framework of interventions and outcomes presented as two dimensions: the rows include interventions with sub‐categories, and the columns outcome domains. Further attributes can be considered and used to filter the results, such as quality of the included studies or characteristics of the included populations. Each cell shows studies which contain evidence on that combination of intervention and outcome. Study characteristics including for example study design, setting and study quality are coded and the evidence can be filtered by these characteristics.

### 3.3 Existing EGMs and or/relevant systematic reviews

There are currently no other EGMs that exist that address this type of intervention, however, it would complement existing EGMs addressing child welfare. There is a recent scoping review that focuses on outcomes for older people that we will use to inform the framework for our EGM (Krzeczkowska et al., 2021).

The EGM framework will inform the inclusion and exclusion criteria of the EGM. Here, we describe the population, intervention, comparison, outcomes (indicators) and study designs for the map.

### 3.4 Stakeholder engagement

The following individuals have agreed to contribute to the project through the advisory group:

Ronald Amanze; Professor Sir Muir Gray—Director of the Optimal Ageing Programme; Iain Lang—University of Exeter; Vicki Goodwin—University of Exeter; Jo Day—University of Exeter; Aideen Young—Centre for Ageing Better; G.J. Melendez Torres—University of Exeter; Dylan Kneale—UCL; Ruth Garside—University of Exeter; Claire Goodman—University of Hertfordshire; Tracey Howe—Cochrane Campbell Global Ageing Partnership; Oliver Rashbrook Cooper—Public Health England; Kelvin Yates—AgeUK Cornwall; Nathan Hughes—University of Sheffield; Debbie Hanson—Sheffield City Council; Laura Abbott—Chilypep; Hannah Fairbrother—University of Sheffield; Kerry Albright—Unicef; Rachel Staniforth—Public Health; Girish Vaidya—Sheffield Children's NHS Foundation Trust; Sally Pearse—Sheffield University. Members of the Only Connect steering group will be invited to contribute throughout the project. The group has local, national and international members from the care sector, local government, academia, schools and leading organisations involved in providing intergenerational activities. Members of the group will also facilitate discussion of the project with older people, people living with dementia and young people with experience of taking part in intergenerational activities.

We will convene three virtual whole project meetings to include stakeholders and advisory group members (during Months 1, 3 and 15) to assist with interpretation and understanding. We will use break out rooms and other methods of sharing ideas and suggestions such as JamBoard to ensure that as many views and perspectives are captured as possible. We will follow these large meetings up with smaller meetings/phone calls if necessary.

Between meetings we will involve people through email, telephone and video conferencing depending on the nature of the involvement and the preference of individuals.

During the stakeholder meeting in Month 1 we shall engage the stakeholder group is informing the development of the framework which will form the matrix for the EGM. Working in small groups, we will encourage participants to identify outcomes and types of intervention. This will be used, along with the wider literature to inform the components of the framework.

### 3.5 Conceptual framework

Our conceptual framework will be informed by the following: the five essential elements of wellbeing described by Nazroo and colleagues [Nazroo] adopted by the Institute for Public Policy Research (IPPR) (Nazroo et al., 2005), the seven outcomes outlined in the Department of Health Social Care Green Paper, Independence, Well‐being and Choice (DOH, 2005) and the six domains identified in which actions are required for child and adolescent health and wellbeing by the World Health Organisation and UNICEF Unicef (WHO, 2020). These models will guide the components of our matrix which will then be further considered by our stakeholders.

**Table:** 

Five essential elements of wellbeing (Nazroo et al., 2005)	Seven outcomes in the social care Green Paper, Independence, Well‐being and Choice (DOH, 2005)	Six domains identified in which actions are required for child and adolescent health and wellbeing Unicef (WHO, 2020)
Resilience	• improved health and emotional well‐being	Good health
Independence	• improved quality of life	Adequate nutrition
Health	• making a positive contribution	Opportunities for learning and education
Income and wealth	• increased choice and control	Securing, safety and a supportive clean environment
Having a role and having time	• freedom from discrimination or harassment	Responsive relationships and connectedness
	• economic well‐being	Realisation of personal autonomy and resilience
	• maintaining personal dignity and respect	

### 3.6 Dimensions

The dimensions of the EGM will be based on an intervention/outcome framework.

The outcomes will be drawn from the engagement with our stakeholders and will be broadly based on the above frameworks.

For the interventions we will use the Depth of Intergenerational Engagement Scale Kaplan, 2004 as the framework for the interventions. These are described below.

#### 3.6.1 The Depth of Intergenerational Engagement Scale

The Depth of Intergenerational Engagement Scale places programmes and activities on a continuum, with points that correspond to different levels of intergenerational engagement, ranging from initiatives that provide no direct contact between age groups (point 1) to those that promote intensive contact and ongoing opportunities for intimacy (point 7). Examples of intergenerational initiatives fitting into each point on the scale are described.

1. Learning about other age groups
  Participants learn about the lives of persons in other age groups, although there is no direct or indirect contact.
  Example: ‘Learning about Aging’ programmes designed to teach youth about aspect(s) of the aging process.
2. Seeing the other age group at a distance
  These initiatives facilitate an indirect exchange between individuals of two or more age groups. Participants might exchange videos, write letters, or share artwork with each other, but never actually meet in person.
  Example: A pen‐pal programme in which youth in an after‐school club exchange letters with residents of a nursing home.
3. Meeting each other
  Initiatives culminate in a meeting between the young participants and older adults, generally planned as a one‐time experience.
  Example: A class of students plan for and visit a local senior center in which all engage in activities during a July 4th picnic.
4. Annual or periodic activities
  Often tied to established community events or organisational celebrations, intergenerational activities occur on a regular basis. Although infrequent, these activities might symbolise intergenerational and community unity and influence attitudes and openness towards additional or ongoing activities.
  Examples: Intergenerational activities at a school on Grandparent's Day, an annual community dance in which youth and older adults are actively involved, and Christmas caroling at assisted‐living homes.
5. Demonstration projects
  Demonstration projects generally involve ongoing intergenerational activities over a defined period of time. Depending on project goals and objectives, the intergenerational exchange and learning can be quite intensive. These initiatives are often implemented on an experimental or trial basis, and frequently depend on external funding.
  Example: A 6‐month pilot programme, sponsored by an agency that provides teen parenthood support services. Senior adults who have successfully raised children are enlisted to mentor and provide support for pregnant and parenting teens.
6. Ongoing intergenerational programmes
  Programmes from the previous category that have been deemed successful and valuable from the perspective of the participating organisations and the clientele are incorporated as an integral part of their operation. This extends to programme and staff development such as preparing individuals to work with populations of various age groups.
  Example: Based on a partnership forged between a senior center, a community youth center, and an environmental education center, senior adults and youth plan and execute the town's environmental improvement campaign. Systems are established to organise numerous projects, train and assign participants, and provide continuing support and recognition.
7. Ongoing, natural intergenerational sharing, support, and communication.

There are times when the intergenerational reconnection theme transcends a distinct programme or intervention. This is evident when the social norms, institutional policies and priorities of a particular site, community, or society reflect values of intergenerational reciprocity and interdependence. Intergenerational engagement takes place as a function of the way community settings are planned and established. In this context, opportunities for meaningful intergenerational engagement are abundant and embedded in local tradition.

Example: A YMCA facility houses a senior citizen center. Older adults and youth participate in a variety of age‐integrated activities. Programmes fitting into all points on this continuum provide positive experiences for interacting with persons in other age groups. However, if the aim is ambitious, such as changing attitudes about other age groups, building a sense of community, enhancing self‐esteem, or establishing nurturing intimate relationships, it becomes important to focus on programmes that fit into levels 4–7 on the scale. Programmes would take place over an extended period of time, would last anywhere from a few months to many years, and would provide extensive interaction opportunities (Kaplan, 2004).

#### 3.6.2 Types of study design

Any study design including systematic reviews, randomised controlled studies, observational studies and evaluations, surveys and qualitative studies. We will also include news items describing intergenerational activities and programmes if they report innovative interventions not otherwise represented within the evidence base.

#### 3.6.3 Types of intervention/problem

Any intervention that seeks to bring older and younger people together intentionally with the purpose of achieving positive health and/or social and/or educational outcomes. These might include reminiscence programmes, buddy systems, storytelling, school‐based interventions and arts‐based interventions. We will use the Depth of Intergenerational Engagement Scale (Kaplan, 2004) as the framework for the interventions.

#### 3.6.4 Types of population (as applicable)

We will include studies that include older adults and children and young people.

No age boundary restrictions will be applied but we will seek information from studies that suggests there is at least one skipped generation between older and younger participants. Studies in which participants are related by family or marriage will be excluded. Inclusion will not be determined by age cut‐offs but by the included studies own definition of ‘older people’ and ‘young people’.

#### 3.6.5 Types of outcome measures (as applicable)

Outcomes may include (but will not be limited to) social isolation, engagement, interacting, perception of people living with dementia, social inclusion, psychological outcomes, depression, anxiety, social skills, self‐confidence, creativity, school performance, relationship building, attitudes, empathy, personal growth, community responsibility, activity levels (physical activities), mood, quality of life, stimulation of memory and mind, digital inclusion (helping people to get online).

Comparator and outcomes will not form part of the criteria for including studies in the EGM since we are keen to explore all of the available evidence

#### 3.6.6 Other eligibility criteria

State any additional eligibility criteria applied to the EGM (e.g., geographical setting).

##### Types of settings

Any setting or context.

##### Status of studies

We will include studies irrespective of their publication status and their electronic availability. We will also include ongoing studies where it is feasible to ascertain that the study will be completed.

### 3.7 Search methods and sources

We will search MEDLINE (via OvidSp), EMBASE (via OvidSp), PsycINFO (via OvidSp), CINAHL (via EBSCOHost, Social Policy and Practice (via OvidSp), Health Management Information Consortium (via OvidSp), Ageline (via EBSCOhost), ASSIA (via ProQuest), Social Science Citations Index (via Web of Science), ERIC (via EBSCOhost), Community Care Inform Children, Research in Practice for Children, ChildData (via Social Policy and Practice), the Campbell Library, the Cochrane Database of Systematic Reviews and the CENTRAL database using terms for intergenerational practices. As we are seeking to identify the richest possible evidence base, we will not place any language or date restrictions on the searches.

We expect that some relevant reports may not be published in academic sources so we will also search for grey literature via relevant organisation websites (e.g., Age UK, Age International, the Centre for Ageing Better, Barnado's, Children's Commission, UNICEF, Generations Working Together, the Intergenerational Foundation, Linking Generations and The Beth Johnson Foundation), conference abstracts via the Conference Proceedings Citation database, and dissertations via ProQuest Dissertations and Theses Global.

To find any published literature not captured by the databases we will review the included studies within relevant systematic reviews and carry out backwards citation chasing (checking reference lists of included studies). We will also check the citations of older key papers (forward citation chasing) and hand‐search the contents of key journals identified during the search process (e.g. Journal of Intergenerational Relationships).

As part of a horizon scanning process we will search Nexus for relevant international news articles about intergenerational practices and Google for relevant reports, blogs, news articles and links to other relevant organisations.

We will set up automated alerts to identify additional relevant literature during the course of the project and use these to update the map if appropriate.

### 3.8 Analysis and presentation

#### 3.8.1 Report structure

The EGM report will provide tabulations or graphs of the number of studies, with accompanying narrative description, by

Intervention category and subcategory

Outcome domain and subdomain

Table of ‘aggregate map’ of interventions and outcomes

Region

Year

Study type

Population subgroups

#### 3.8.2 Filters for presentation

We will use EPPI‐Reviewer software for data extraction and coding and to generate the online evidence map. The map will be interactive so that users can click on cells within the matrix to show a list of the relevant studies and on study names to access the study or a reference and database link for the study.

Findings will also be presented in a descriptive report that will summarise the evidence. The report will include a description of the methods used, the spread and concentration of evidence across intervention and outcome categories and will highlight important evidence gaps and trends.

In addition to the interventions and outcomes, the following filters will be coded:

Characteristics of the participants that the intervention is aimed at:

- Mental health difficulties (*both*)
- Physical health difficulties (*both*)
- Minority groups (*both*)
- Low socioeconomic status (*both*)
- Unemployed (*both*)
- Educational needs (*both*)
- Social isolation (*both*)
- Age category of the *children/young people*—0–5years, 6–12 years, 12–18 years, 19–30 years.
- *Children* experiencing childhood adversity
- *Older peopl*e with cognitive impairment

Contextual factors:

- Country/region—country of the first author
- Setting—where the intervention happened, for example, in school, care home, retirement village, university/higher education, shared facility, day care centre, hospital, assisted living centre or community setting

Study design factors:

- Study design—randomised controlled trials, non randomised controlled trials, interrupted time series, controlled before and after studies, observational studies, qualitative studies, mixed methods and systematic reviews

Focus of the intervention (the activities involved in the intervention):

- Education—where older or younger generations teach the other generation a skill or share educational knowledge
- Art—generations share in arts or crafts
- Music—generations share musical activities or teach a musical skill
- Interaction—interaction between the generations like conversation, spending time/communication, helping tasks

#### 3.8.3 Dependency

Each entry in the map will be a systematic review or a primary study of effectiveness. The final EGM will identify the number of studies covered by the map in each sector or subsector. We will link all publications from the same study (e.g. protocols, secondary analyses). We will include all relevant systematic reviews and primary studies irrespective of whether there is overlap between reviews and studies. Similarly studies with multiple interventions or multiple outcomes may appear multiple times within the map.

### 3.9 Data collection and analysis

#### 3.9.1 Screening and study selection

The titles and abstracts of records identified by bibliographic and supplementary search methods will be screened against inclusion criteria by two independent reviewers looking for reasons for exclusion. The full text of records retained at this stage will be retrieved and screened for inclusion against the inclusion criteria using the same process. All included studies will form a master library using EndNote X8 and will form the basis for the study selection processes in the REVIEW phase of the project.

#### 3.9.2 Data extraction and management

Data extraction will be undertaken by one reviewer and checked by a second with any inconsistencies identified and resolved through discussion. The data extraction tool will be modified and tested through stakeholder and advisor consultation and piloting the process. The tool will be informed by the research question and the structure of the map.

Final decisions on the data we will collect will be made with stakeholder involvement but is likely to include data on study characteristics, geographical location, setting, population (age, gender, health condition/status, equity characteristics), intervention (type, mode of delivery, setting) and outcomes.

We will use the PROGRESS‐Plus framework (O'Neill et al., 2014) to identify studies that have measured effects of interventions by gender or other health inequalities.

#### 3.9.3 Tools for assessing risk of bias/study quality of included reviews

The map will include any study design (systematic reviews, randomised controlled studies, non‐randomised controlled studies, comparative studies, observational studies, evaluations, surveys and qualitative studies) in addition to ongoing studies. The design of the included studies will be described and indicated on the map. We will not undertake quality appraisal of the individual studies. We will use study design to identify areas that need more research or more robust research bearing in mind that not all research is equal and that some research designs that are considered more robust can still be weak or poorly conducted.

#### 3.9.4 Methods for mapping

EPPI reviewer will be used for data extraction and to code and produce the EGM.

## CONTRIBUTIONS OF AUTHORS

Content: ERC is a socially engaged creative practitioner based in Falmouth and Project Officer at The Sensory Trust where she works on the Creative Spaces in the Community Project. This project uses nature and outdoor spaces to encourage older people with dementia to become more active, build social networks and foster independence. Previously she founded the multi‐award winning Penryn Memory Café and led a memory café in York for 2 years whilst at University. She has recently completed the International Certificate in Intergenerational Practice provided by Generations Working Together and the University of Granada. SC is Commissioning Manager at NHS Kernow Clinical Commissioning Group and has an interest in the role of intergenerational programmes and activities in health and social care. RS is an advanced public health specialist at Cornwall Council with an interest in the role of intergenerational programmes and activities in health and social care specifically in relation to the mental health of older adults. JB is an expert in the mental and social wellbeing of children and young people and also has expertise in evidence synthesis methodology.

EGM methods: JTC is an expert in evidence synthesis and health policy research. She is cochair and editor of the Ageing Group of the Campbell Library and codirector of the Cochrane Campbell Global Ageing Partnership. RW is an expert in evidence synthesis methods. FC is editor of the Children and Adolescent Group of the Campbell Collaboration. She has over 20 years of experience in evidence synthesis and is leads a short course in scoping, mapping and EGM reviews. AB is providing methodological advice and support for the reviewing process.

Information retrieval: MR is an information specialist with experience in health services research, methods editor for the Ageing Group of the Campbell Library and a member of the Campbell Information Retrieval Methods Group. AS is a Senior Information Specialist, with extensive experience of literature searching and information management for systematic reviews and other types of evidence syntheses on a wide range of topics. Anthea has experience of literature searching for a wide range of health and social care topics, including integrated care, art therapy, and quality of life in children with speech and language difficulties. Anthea is the joint lead of a module on systematically reviewing the research literature for postgraduate students, and the joint author of the textbook ‘Systematic Approaches to a Successful Literature Review’ 2nd Edition published by Sage in 2016. Anthea is also the Reviews Editor for Health Information and Libraries Journal.

## DECLARATIONS OF INTEREST

ERC, members of our advisory group and members of the Only Connect steering group are involved in the delivery of intergenerational activities and programmes.

## PLANS FOR UPDATING THE EGM

Once completed the evidence gap map will be updated as resources permit.

### SOURCES OF SUPPORT

Internal sources

- No sources of support provided
  External sources
- National Institute for Health Research (NIHR) Evidence Synthesis Programme NIHR 133097 and NIHR 133172, UK

The evidence gap map is funded by the National Institute for Health Research (NIHR) Evidence Synthesis Programme NIHR 133097 and NIHR 133172 and supported by the National Institute for Health Research (NIHR) Applied Research Collaboration South West Peninsula. The views expressed are those of the author(s) and not necessarily those of the NIHR or the Department of Health and Social Care.

## Supporting information

Supporting information.
